# The Action of Mercury in Children

**Published:** 1871-11

**Authors:** W. L. Nichol


### The Action of Mercury in Children.

By W. L. Nichol, M.D.

At a late meeting of the Medico-Chirurgical Society of Edinburgh

Dr. Stephenson read a paper on this subject, of which the following is a brief abstract:

He remarked that recent physiological experiments tended to prove that mercury had no direct stimulant action on the biliary secretion; but, so long as these experiments were made only on healthy organs, they could never demonstrate that it had no effect on the pathological conditions which were interfering with the performance of the function, and the result of such experiments would never overturn its use after the manner of a rational expiricism. An analysis of its action in the intestinal affections of children showed that its good effects could be explained on other grounds than its mere cholagogue action; and, although this theory of its action was erroneous, mercury was still of service. Its use, however, should be limited almost wholly to its action as an occasional purgative. When given to produce its constitutional effects, the constitutional type or diathesis greatly determined its action. It was in subjects most liable to those external diseases where mercury was of service when applied externally, that internal affections were most likely to be omenable to its constitutional use. From the consideration of its internal use, illustrated by cases, the following conclusions were drawn:

1. Mercury may be employed to influence the constitution without any injurious effect on the general health.

2. To obtain its therapeutic action, it is not necessary to produce its visible physiological effects; it becomes injurious so soon as these are manifested.

3. It is an error to suppose that children are less susceptible to its influence than adults; its effects are not to be looked for in its action on the mouth, but in its depressing effects, and in deterioration of the blood.

4. It should be used only so far as is necessary, and in order to stimulate the nutritive changes in the tissues, not in the character of the constituents of the blood; for this purpose it should be used as a whip or spur only—that is occasionally and at intervals, not continuously throughout the whole course of the disease.

5. Its use is modifying acute inflammatory action is very limited, and requires further observation; but there is no question as to its power over the products of inflammation, in starting the processes of resolution and absorption where these have been arrested; and, where other remedies fail in producing a change on morbid nutrition, it does in certain cases succeed in promoting a return to healthy action.

6. No number of cases improperly treated by mercury, no number of "constitutions shattered by its abuse," no number of instances where disease has been cured without it, can in any way invalidate the results of its effects, where it has cured where other remedies have failed, or lessen in any measure the position here defended of a judicious use of the medicine. In infantile syphilis. the author has the greatest confidence in the good effects of mercury, but limited its use, as in other diseases, and stopped it whenever amelioration in the symptoms was observed, renewing it, if necessary, and not giving more than one or two grains of gray powder daily for a fortnight.

Dr. George Balfour remarked on the interesting and practical nature of the paper, and agreed with the author's views on the treatment of chronic cases of pneumonia with mercury.

Dr. Bennett had considerable difficulty in criticising a paper which discussed such important topics, containing as it did much assumption and little fact. What the author understood by "rational empiricism" he did not know; but, for his own part, he would define it as experimental theapeutics, based on a knowledge of anatomy and physiology. Dr. Stephenson maintained that mercury was not a cholagogue, and had no influence whatever in causing bilious stools in children. He also maintained that jaundice in children yielded to the simplest treatment. As he (Dr. Bennett) agreed with the author on these points, he need not dwell upon them. But Dr. Stephenson supported the ideas of those who maintained calomel to be a sedative, and had stated that eight or ten grains of calomel in an adult, and three or four grains in a child, when mixed with sugar and placed on the tongue, at once checked obstinate vomiting. In India, much larger doses, viz., thirty grains, had been recommended as a sedative in dysentery and cholera. He (Dr. Bennett) believed such practice to be of no benefit, but, on the contrary, to be dangerous. If placed on the tongue and not swallowed, mercury was likely to produce violent stomatitis and salivation, such as occurred in the man Holden, whom he had presented to the Society at a former meeting. Dr. Stephenson also supported the employment of mercury in the syphilis of young infants. He (Dr. Bennett) did not consider it to be of any service in such cases, nor in those of diphtheria. Further, Dr. Stephenson called it a stimulus to the tissues, said it acted as a whip or spur in chronic inflammatory diseases, and had given three cases as examples. They were quite insufficient to support such a proposition, which, if extensively followed, would, in his opinion, be productive of great mischief. Dr. Stephenson had stated that mercury increased cell-growth and caused proliferation of cells. But did he possess any facts or series of preparations capable of supporting this assertion? Such an idea also appeared to be opposed to what was said in the first part of the paper; viz., that it caused anæmia and destruction of tissue. What was at present required in therapeutics was an effort to distinguish between the supposed action of remedies and the spontaneous cure of diseases. Wherever this, in recent times, had been carefully carried out, it had been shown that in many diseases violent remedies, such as bleeding and mercury, were opposed to a rapid recovery. He referred to the diminution in the mortality and duration of pneumonia, since antiphlogistics had been abandoned; and hoped before long to convince the Society, by a tubular view of cases comprising the bad as well as the good ones, that in hepatic disorders mercury could only be considered as useless or injudicious.

Dr. Angus Macdonald agreed with much that Dr. Bennett had said; but, though he set out in practice a pupil of Dr. Bennett's, with a profound skepticism as to the use of mercury, he found himself, by experience, compelled to use it in many cases, from having seen the advantage of its use.

Dr. George Balfour, in answer to Dr. Bennett, showed that mercury, in acting as a destructive agent on tissue, acted chiefly on new and morbid tissue, and by its destructiveness did good. He also pointed out how large doses of calomel acted as mere irritant purgatives, and were not absorbed, while very small doses were absorbed by the system.

Dr. Stephenson, in reply, stated that, in his opinion, Dr. Bennett had not appreciated the points illustrated by his cases; and that Dr. Bennett did not distinguish between the use and the abuse of a remedy.—*British Medical Journal, March* 25, 1871.—*Nashville Med. Journal.*

